# Challenges facing translational research organizations in China: a qualitative multiple case study

**DOI:** 10.1186/1479-5876-11-256

**Published:** 2013-10-13

**Authors:** Laixin Zhou, Ying Li, Hayden B Bosworth, John Ehiri, Changkun Luo

**Affiliations:** 1 College of Preventive Medicine, Third Military Medical University, Chongqing, China; 2 Department of Social Medicine & Health Service Management, Third Military Medical University, Chongqing, China; 3 Department of Medicine / General Internal Medicine, Duke University, Durham, NC, USA; 4 Division of Health Promotion Sciences/Global Health Institute, Mel & Enid Zuckerman College of Public Health, University of Arizona, Tucson, Arizona, USA; 5 Third Military Medical University, No. 30 Gaotanyan Road, Chongqing, Shapingba district 400038, China

**Keywords:** Translational medicine, Translational research organization, Translational medical center, Case study

## Abstract

**Background:**

Translational medicine is attracting much attention worldwide and many translational research organizations (TROs) have been established. In China, translational medicine has developed rapidly, but faces many challenges. This study was aimed at exploring these challenges faced by emerging TROs in China.

**Method:**

A qualitative, multiple case study approach was used to assess the challenges faced by TROs in China. Data were collected between May and August 2012.

**Results:**

Eight cases were identified. Overall, four themes that characterized TROs in China emerged from analyses: 1. objectives, organizer, and funding resources, 2. participating partners and research teams, 3. management, and 4. achievements. All TROs had objectives related to translating basic discovery to clinic treatment and cultivating translational researchers. In terms of organizer and funding resources, 7 out of 8 TROs were launched only by universities and/or hospitals, and funded mostly through research grants. As for participating partners and multidisciplinary research teams, all but one of the TROs only involved biomedical research institutions who were interested in translational research, and characterized as clinical research centers; 7 out of 8 TROs involved only researchers from biomedicine and clinical disciplines and none involved disciplines related to education, ethnicity, and sociology, or engaged the community. Current management of the TROs were generally nested within the traditional research management paradigms, and failed to adapt to the tenets of translational research. Half of the TROs were at developmental stages defined as infrastructure construction and recruitment of translational researchers.

**Conclusions:**

TROs in China face the challenge of attracting sustainable funding sources, widening multidisciplinary cooperation, cultivating multi-disciplinary translational researchers and adapting current research management to translational research. Greater emphasis should be placed on increasing multidisciplinary cooperation, and innovating in education programs to cultivate of translational researchers. Efforts should be made to reform research management in TROs, and establish sustainable funding resources.

## Introduction

Translational medicine/research/science has become an issue of increasing importance to scientists and governments around the world. The history of translational medicine can be traced back to 1966, when the notion of “From bench to bedside” was presented in *Bioscience*[1]. The concept of “bench to bedside” was further raised in 1992 in the journal of *Science*[2], and in 1996s, the concept of “translational research” was presented in the *Lancet*[3]. Dr. Elias Zerhouni, former Director of the United States National Institutes of Health (NIH) defined the term "translational medicine" for the first time in 2003 [4]. Scientists have conducted successful studies in many areas of translational medicine [5-8] and the scope of translational medicine/research/science is evolving and enlarging [9-17]. The overall goals of the “translational” approach are to translate between basic science research and practice in novel diagnostics/prognostics/therapeutics for patients as well as community prevention approaches, regulations and public policies to influence basic research [17,18]. Translation research is a biomedical research translation continuum that includes 4 practical landmarks (basic science discovery, proposed human application, proven clinical application, and clinical practice) which corresponds to the three translation chasms often referred to as T1 (defined as from basic science to clinical science), T2 (defined as from clinical science to clinical practice), and T3 (defined as from clinical science to health improvements) [13]. The complete translation continuum is a complex process and takes an average of 17 years for research evidence to reach clinical practice [11]. Given the time and complexity of translating findings into care, there is a continued need to promote the concept of translational medicine among clinicians, basic science researchers, biotechnologists, politicians, ethicists, sociologists, and investors and to further improve efficiency of these translational processes [19].

The past three decades have witnessed tremendous advances in China in terms of living conditions, food, nutrition, and health systems reform [20]. However, while the economy grows and the society rapidly transforms, the healthcare system still faces multiple problems [20]. Communicable diseases such as hepatitis B virus, tuberculosis, and HIV remain a heavy burden, while at the same time, chronic diseases, including cancer, cardiovascular disease, hypertension, and diabetes mellitus have emerged as leading causes of mortality and disability in China [20]. Along with accelerating economic growth, China is experiencing rapid population aging [21]. Soaring health needs and limited health resources as a result of an aging population, shifts in disease patterns, and failure of the health market indicate that it is urgent for China to develop strong translational medicine programs to move efficacious interventions into the health care delivery system. Fortunately, with the support of governments and scientists, translational medicine in China has been developing rapidly in recent years [20,22-28].

To promote translational research, it is required that nations set up translational research organizations (TROs) and integrate them into the larger network of bench to bedside translation continuum [28]. In countries with developed translational medicine, such as the U.S., the government has funded a network of translational science institutes or centers from national to state levels across the country [29]. In China, many TROs have been established or have been in the process of development since 2009. However, translational medicine has met a lot of challenges in China. The aim of this study was to understand the challenges facing translational medicine in China in order to gather information that may inform the creation of more efficient organizational structure, functions, and performance of Chinese TROs.

## Method

Case studies have a major function in generating hypotheses and building theory [30]. Usually, better understanding of a phenomenon is gained by conducting multiple case studies [31]. Multiple case studies enable the researcher to explore differences within and between cases in order to deeper insight into phenomena of interest [31,32]. A qualitative multiple case study design was used to examine the construction and performance of TROs in China and was conducted between May and August 2012.

### Definition of TROs

In this study, TROs refers to institutes explicitly with the title of 'Translational Medical Center’; these centers have a multidisciplinary research group (basic and clinic researchers, statisticians, psychologists, educators and sociological researchers) who work as a team aimed at translating basic discovery to disease diagnosis, treatment, and prevention, or population health promotion, while simultaneously cultivating translational researchers.

### Case selection

TROs were selected if they met the following criteria: (1) had the designation of a translational medical center, translational research center or translational medical research center; (2) had a multidisciplinary research team including representatives from basic medicine, clinic medicine, statistics, psychology, and social sciences; (3) aimed at translating basic discovery to diagnosis, clinic treatment, prevention, or population health promotion, (4) aimed at cultivating translational researchers; (5) had representations from hospitals and/or universities; (6) had representations from a wide economic and geographic spectrum throughout China, and (7) established before January 2012.

### Data collection method

Data was triangulated by integrating interviews and review of secondary sources.

(1) Interviews: The purposive sampling technique (based on the afore-mentioned inclusion criteria) was used to select TROs for interview. We used an interview guide to collect data on year of establishment, objectives of TROs, funding resources, research teams (including multidisciplinary and institutional), management teams and approaches, and achievement (platform establishment, translational scientist cultivation, funding, cooperation, and translational research outcomes).

(2) Review of secondary sources: Google, Google Scholar, and PubMed were used to search related literature and reports published up to May 31st, 2012, on Chinese TROs. Search terms included translational research center in China, translational medical research center in China, and translational research institution.

(3) Meeting reports review: We collected mainly documents from the 2012 Sino-American Symposium on Clinical and Translational Medicine (SAS-CTM).

### Data analysis

We combined qualitative data from all sources and analyzed them using qualitative content analysis method [30]. The analysis included three phrases: preparation (reviewing the data), organization (coding, categorizing, and triangulating the data), and cross-case synthesis. The main themes that emerged following data coding included date of setup for the TROs, objectives of the TROs, organizer, funding sources, participating partners and multidisciplinary research teams, management (team, project management, cooperation, and funding), and achievements (infrastructure establishment, research team establishment, translational research, service, and outcomes including publications, awards, intellectual properties, products, and international communication) (Additional file 1).

## Results

### General information on cases

**Figure 1:**
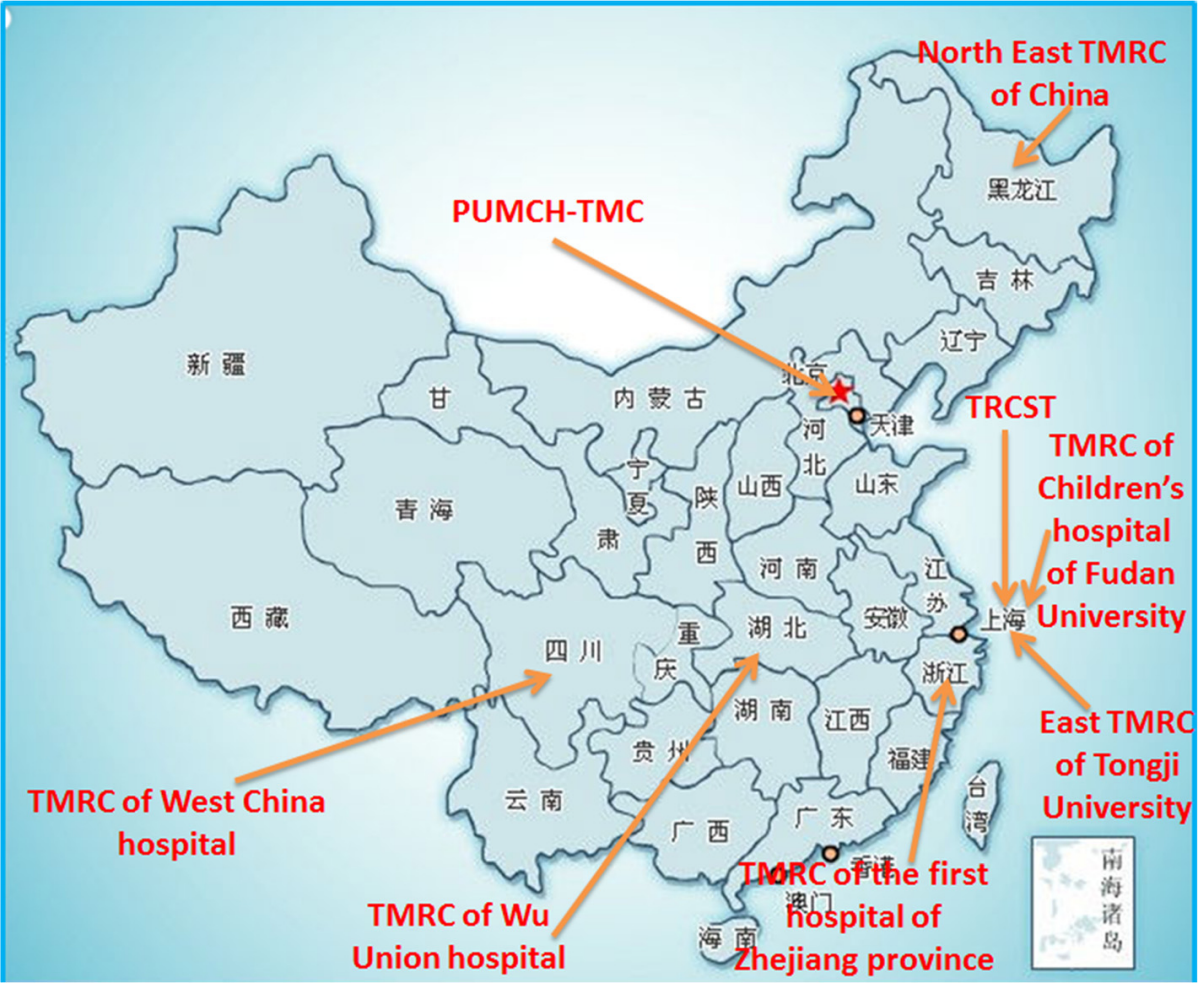
**Distribution of cases in this study.** This figure is a description of the distribution of cases in this study.

In total, 8 TROS were identified. They included TROs distributed in the north, south, west, and east of China, in both high and low income regions (Figure 1). Characteristics of the 8 TROs are presented in Table 1. All of the TROs were established between 2008 and 2011: two in 2008 and 2009, respectively, four in 2010, and two in 2011. Among them, one was set up as an independent college (College of Translational Medical Research (CTMR) of the first hospital of Zhejiang province); the rest were set up as Translational (Medical) Research Centers. Four cases were located in teaching hospitals affiliated with universities, three were located in universities, and one was located in a non-teaching hospital (Table 1). Data from CTMR in the Zhejiang province was collected only by interview. In three cases, data was collected by using both interviews and reports. In three cases, data were obtained from reviewing documents from both the 2012 Sino-American Symposium on Clinical and Translational Medicine (SAS-CTM) and published reports; finally, data from Peking Union Medical College Hospital (PUMCH) was collected by a study of literature and a review of reports (Table 1).

**Table 1:** General information of cases

**TROs**	**Year of construction**	**Location**	**Methods of data collection**
PUMCH-TMC	2010	Teaching Hospital	Literature and report [33,34]
CTMR of the first hospital of Zhejiang province	2010	University	Interview
TMRC of Children’s hospital of Fudan University	2008	Teaching Hospital	Interview and report [35]
TRCSR in Shanghai Ninth hospital	2011	Hospital	Document review and report [36]
TMRC of North East of China	2010	University	Document review and report [37]
East TMRC of Tongji University	2010	University	Document review and report [38]
TMRC of Wuhan Union Hospital	2011	Teaching Hospital	Interview and report [39]
TMRC of West China Hospital	2009	Teaching Hospital	Interview and report [40]

Notes: TROs refers to translational research organization; PUMCH refers to the Peking Union Medical College Hospital; TMC refers to translational medical center; CTMR refers to College of Translational Medical Research; TRCSR refers to Translational Research Center of Stem Cell and Regenerative Medicine.

### Objectives, organizer and funding sources of TROs

All of TROs focused on facilitating translation between clinical and basic researchers (promoting clinic-basic multidisciplinary cooperation) and cultivating translational scientists. However, few TROs explicitly stated any consideration about translating research into health policy and public health (Table 2). Except for one TRO, which was launched through a joint effort of the government and a university (Pudong district and Tongji University), the rest were launched by universities and/or hospitals. As for funding sources, most of the TROs were funded by partners within TROs or through research grants. Only East TMC of Tongji University was supported by the local government but not the central government. CTMR of the Zhejiang province was awarded funds from social services agencies (Table 2). It was notable that no TROs had funding from industry.

**Table 2:** **Objectives**, **organizer and funding resources of TROs**

**TROs**	**Objective**	**Organizer**	**Funding resources**
PUMCH-TMC	To provide a robust platform for translational medicine research programs (to provide consulting services and technical support to researchers, create more collaborations and partnerships) and provide post-graduate education. With consistent effort, the PUMCH-TMC aims to become a national and international translational medicine institute	Hospital	No data
CTMR of the first hospital of Zhejiang province	To assist cooperation between basic and clinical research, provide support for translation and cooperation, and innovate management to realize cooperation of research groups	University	Funded by social organizations and institutes in CTMR
TMC of Children’s hospital of Fudan University	To be become the national or Asian center of child medicine and medical teaching and research and to facilitate the integration of basic and clinic research	University	Funded by research grants and institutes in TMC
TRCSR	To assist multidisciplinary cooperation, cultivate translational scientists, and provide a platform of domestic and international translational research in the field of regenerative medicine	Hospital	Funded by research grants and institutes in TRCSR
TMC of North East of China	To integrate basic research, clinic medicine, and bio-industry; cultivate translational scientists; form a translational medical research network; integrate preclinical medicine, clinical medicine and drug development; establish open and cooperative networks in Northeast China; and develop medicine, disease diagnoses, and strategies for treatment	University	Funded by research grants and institutes in TMC
East TMC of Tongji University	To integrate government, enterprise, education, research, and medicine; promote integration of basic and clinic care; prioritize the development of translational medical research as the primary task, adhering to the operation mode of “political, industry, education, research and medical field”; and fully integrate with advanced clinical and scientific research resources	Government of Pudong district and Tongji university	Funded by research grants and local government
TMC of Wuhan Union Hospital	To bridge basic, clinical, and bio-industry in order to promote new technology, products, and drugs; promote multidisciplinary cooperation; and cultivate translational scientists	University	Funded by research grants and institutes in TMC.
TMC of West China Hospital	To promote cooperation between basic research and clinical care, support outcome translation, insist on research-education-industry cooperation, and encourage this center and industry to participate in translational research by a joint-stoke model.	University	Funded by research grants and institutes in TMC

Notes: TROs refers to translational research organization; PUMCH refers to the Peking Union Medical College Hospital; TMC refers to translational medical center; CTMR refers to College of Translational Medical Research; TRCSR refers to Translational Research Center of Stem Cell and Regenerative Medicine.

### Organizations of TROs

We collected data about collaborating partners and multidisciplinary research teams involved with the respective TROs. As for partners, East TMC of Tongji University included a university, a hospital (Shanghai East Hospital), and government organizations such as the Pudong New District and Technology Committee and the Pudong New District Health Bureau. The remaining seven TROs were comprised of only research institutes and hospitals. Regarding multidisciplinary research teams, only TMC of Wuhan Union Hospital included public health; the other TROs involved researchers from biomedical and clinical disciplines, with no public health involvement. None of the TROs involved disciplines related to education, behavioral sciences, and sociology, or engaged the community (Table 3).

**Table 3:** Organization of TROs

**TROs**	**Partners**	**Research team**
PUMCH-TMC	Institute of Basic Medical Science of the CAMS, PUMC, and Institute of Pathogen Biology of the Chinese CAMS	Fifty academicians from areas of regenerative therapy using stem cells for central nervous system diseases, the integrated study of the clinical features and molecular mechanisms of pancreatic cancer, HIV/AIDS, chronic metabolic diseases (diabetes, rheumatologic diseases, and the diagnostic and therapeutic use of allergens)
CTMR of the first affiliated hospital of Zhejiang province	Department of neuropsychological disease, gastrointestinal tumor, cardiovascular disease, reproductive health, kidney disease, visual reconstruction, hemopoietic disease and stem therapy, and laparoscope technology	Twenty-thirty PIs and research groups from the above departments
TMC of Children’s Hospital of Fudan Univeristy	Children’s Hospital, Biomedicine Research Institute, and the Institute of Developmental Biology and Birth Defects	Ten to 15 research groups with 15–25 principle investigators (PIs), 25–30 research assistants from the areas of pediatrics, developmental biology, and birth defects
TRCSR	Shanghai Ninth People’s Hospital	Researchers in tissue engineering and stem cell therapy
TMC of North East of China	Dalian Institute of Chemical Physics, Chinese Academy of Sciences, and Harbin Medical University	Researchers from the Department of Biotechnology of Dalian Institute of Chemical Physics including Drug Separation, Analytical Chemistry, Medicinal Biotechnology, Biomaterials and Biotechnology, Energy, and Environmental Biological Technology
East TMC of Tongji University	Pudong New District and Technology Committee, Pudong New District Health Bureau, Tongji University, and Shanghai East Hospital	Forty-one staff members, including 36 full-time personnel and 15 PIs in the area of cardiovascular diseases, inflammation and tumors, psychosomatic diseases, and trauma emergency treatment
TMC of Wuhan Union Hospital	Huazhong University of Science and Technology, Wuhan comprehensive drug research and develop platform, innovative biomedical-clinic research platform, and Wuhan Biolake	Clinical medicine, basic medicine, public health, pharmacy, and bioscience
TMC of West China Hospital	West China hospital	A total of 1,000 researchers from clinical medicine, pharmacy, biotechnology, chemistry, and materials science

Notes: TROs refers to translational research organization; PUMCH refers to the Peking Union Medical College Hospital; TMC refers to translational medical center; CTMR refers to College of Translational Medical Research; TRCSR refers to Translational Research Center of Stem Cell and Regenerative Medicine.

### Management of TROs

We focused on management teams and research groups, funds, and projects. In terms of management teams, all but one TRO (CTMR of the first affiliated hospital of Zhejiang Province) had a director, vice director, and an administration office. In addition, all TROs had various committees, including a management committee, an academic committee, and a consulting committee (Table 4). Regarding team management, all TROs paid most attention to recruiting researchers, setting up principal investigators (PI), and nurturing translational researchers (through continuing education, joint-cultivation of graduates, training, and academic visits). There was no specific strategy to cultivate multi-disciplinary translational researchers and there were no Master's or PhD degree programs in translational medicine in any of the TROs (Table 5).

**Table 4:** Management team in TROs

**TROs**	**Leader**	**Committee**	**Others**
PUMCH-TMC	Director of TMC	Expert committee: Two honored directors, 1 director, five associate directors, and forty-three committee members	No data
CTMR of the first affiliated hospital of Zhejiang province	One dean and one vice-dean	Expert committee: Participants from the university, basic medicine, and the hospital	Management office: To carry out administration
			PI: One PI in each research area
TMC of Children’s hospital of Fudan University	One director, two associate directors	Academic committee, human resource committee, resource and technological platform management committee	Administration office: One-two secretaries
TRCSR		Expert committee: Overall planning, projects and budget review, stage and final assessment)	Office of Translational Research Center: Project management, professional staff training in translational medicine, website management
		Organizing committee: To establish an efficient and timely information network and provide efficient services; to assist the expert committee with reviewing and revising projects and budgets; to coordinate project implementation	
TMC of North East of China	One director	Management committee: One honorary director, one director, one vice-director, ten committee members	No data
East TMC of Tongji University	One director	Academic Committee, Management Committee, and Advisory Panel (composed of representatives from “political, industry, education, research, and medical fields”)	Research Center Management Office: To be responsible for day-to-day management and liaison work.
		PI: One PI in each research area	
TMC of Wuhan Union Hospital	One director and four deputy directors	Academic committee: One director, several deputy directors, and a committee	Management Office: One director and two secretaries, to be responsible for day-to-day management
TMC of West China Hospital	One director	No data	No data

Notes: TROs refers to translational research organization; PUMCH refers to the Peking Union Medical College Hospital; TMC refers to translational medical center; CTMR refers to College of Translational Medical Research; TRCSR refers to Translational Research Center of Stem Cell and Regenerative Medicine.

**Table 5:** Management of TROs

**TROs**	**Researcher management**	**Project management**	**Cooperation**	**Others**
PUMCH-TMC	Recruiting part-time or full-time translational scientists at home locally and abroad	No data	Cooperating with the University of California in San Francisco to work on international translational medicine collaborations	No data
CTMR of the first affiliated hospital of Zhejiang province	Recruiting part-time or full-time translational scientists at home and abroad, setting up PI	Project selection by expert committee and PI (CTMR provides platform), shared resource seed funds for selected projects	Exploring cooperation with institutes outside CTMR by setting up research alliances, joint laboratories, and shared grants. Setting up policies about publications and intellectual property across institutes. Setting up cooperation with the University of California, Los Angeles	Performance evaluation: CTMR organizes experts at home and abroad to evaluate TRO performance every 3 years according to a list of fixed goals
				Shared source management: Open to all researchers freely, paid for the source which bought by researcher group themselves before.
				Funds: Attract funding from provincial and national governments
TMC of Children’s hospital of Fudan University	Recruiting part-time or full-time translational scientists at home and abroad, exchanging translational researchers, jointly cultivating graduates, setting up PI	No data	Training of translational researchers	Funds: Attract government funds
TRCSR	Arrange qualified medical professional and management personnel to attend related courses in a planned and purposeful manner	To organize the expert committee for the planning, discussion, revision and evaluation of each project, and coordinate the project implementation; to establish an information network for the project; to assist in the application of clinical trial; to organize the investigation, application, and screening of the project, and submit them to the expert committee for further consideration.	No data	3-year Goals: Recruit translational scientists, train translational researchers, translate 3 to 5 projects to clinical practice, build up a Sino-Australian collaborative laboratory
TMC of North East of China		No data	No data	No data
East TMC of Tongji University	Developing translational researchers by visiting, continue education, academic cooperation, joint-cultivating PhD	No data	External Cooperation: Mutual visits, joint training program for doctoral students, advanced study of staffs, scientific research cooperation and co-host international academic conferences	No data
TMC of Wuhan Union Hospital	Setting up PIs policy and academic committee review the PIs	No data		Researchers management:
TMC of West China Hospital	Setting up policy for recruitment of translational scientists	No data	Management of cooperation and intellectual refers to policy/approach in the West Sichuan University	Funds: Application of research grants

Notes: TROs refers to translational research organization; PUMCH refers to the Peking Union Medical College Hospital; TMC refers to translational medical center; CTMR refers to College of Translational Medical Research; TRCSR refers to Translational Research Center of Stem Cell and Regenerative Medicine.

Overall, TROs in China used both internal and external collaborators. As for internal collaboration, one TRO (TMC of Children’s Hospital of Fudan University) had a policy on internal cooperation across institutes and focused on sharing resources and intellectual property, as well as on joint publications. Two TROs (PUMCH-TMC and CTMR of the first affiliated hospital of Zhejiang province) started international cooperation with universities in the U.S. to educate translational researchers. Two TROs (CTMR of the first affiliated hospital of Zhejiang province and TMC of Children’s Hospital of Fudan University) attracted local government funding (Table 5).

Most TROs, however, had no specific strategies for education programs related to cultivation of translational scientists, faculty promotion or performance assessment, promotion of multidisciplinary cooperation inside and outside, or evaluation of outcomes of the different stages across translational continuum. Only the CTMR of the first affiliated hospital of Zhejiang had a policy about performance evaluation, organizing experts locally and abroad to evaluate TRO performance every 3 years according to a list of fixed goals (Table 5).

### Achievements of TROs

A majority of TROs are at the beginning stage, few had experienced substantial achievements as TROs. Their main achievements had been predominantly infrastructure construction and recruitment of translational researchers. Three TROs carried out international collaboration. Some TROs had created products such as implantable ventricular assist device (VAD), published papers (Forty-three papers in SCI journals), and/or received awards or patents. Only TRCSR provided a service for training translational researchers in 2012 (Table 6).

**Table 6:** Achievements of TROs

**TROs**	**Infrastructure**	**Researcher group**	**Translational research**	**Others**
PUMCH-TMC	Platform establishment	Researcher recruitment mode	No data	International cooperation: Starting translational researcher cultivation with UCSF
CTMR of the first affiliated hospital of Zhejiang province	No data	No data		No data
TMC of Children’s hospital of Fudan University	10 related bio-medical labs were established	Recruited 18 international famous experts in molecular genetics, neurodevelopment and molecular biology		No data
TRCSR	construction of the state of art GMP standard laboratory	No data	Select the first 15 translational projects, Independent design and commercialization of reagents for embryo verification and warming	Service: successfully held the first and second phase of training workshop in March and May, 2012; held the golden symposium in April 2012.
TMC of North East of China	Six platforms were established	No data	Studying on cardiovascular diseases, brain glioma, Qualifying the hospital drug preparations, Screening and development of new drugs, micro RNA delivery system.	No data
East TMC of Tongji University	Currently, six key technology functional units have been established. Two more functional units has been launched in 2012 and will be established	No data	It achieved great success in the development of implantable ventricular assist device (VAD) and the study of its treatment in end-stage heart failure; Undertaken 35 authorized clinical drug trial from enterprises.x	Research funds: 17.35 million Yuan research funds
				Outcome (Papers, awards, patent): Forty-three SCI papers were published as the first author or corresponding authors; the Center has won 4 technology achievement awards; Submitted 3 applications of. National invention patent.
				International cooperation: Established cooperation with 13 research institutes
TMC of Wuhan Union Hospital	Established cooperation with drug enterprises	No data	No data	Outcome: invented *Glideslope*
TMC of West China Hospital	Established 6 technological platforms for the drug research and translational research	Organized researcher groups	No data	International cooperation: NIH-TMC of West China Hospital international Symposium in 2011.
				Signed contract with several drug enterprises,

Notes: TROs refers to translational research organization; PUMCH refers to the Peking Union Medical College Hospital; TMC refers to translational medical center; CTMR refers to College of Translational Medical Research; TRCSR refers to Translational Research Center of Stem Cell and Regenerative Medicine.

## Discussion

Translational medicine has been supported by the Chinese government. The Chinese government has encouraged integrating industry-academic-research [22]. The 12th Five-year Socio-economic Development Plan (2011–2015) in China emphasized improvement of medical research through developing translational research [20]. The *Medium and Long Term Science and Technology* [*S&T*] *Development Plan* (*2012*–*2030*) issued in 2013 also included emphasis on translational medicine [41]. However, while the Ministry of Science and Technology has just supported a project for building a platform of clinical resources [22], translational medicine in China is still in its early stages. This case study revealed that TROs face many challenges including: 1) They were launched by bio-medical research institutions and are characterized as actual clinic research centers; 2) TROs are not integrated with educational programs that are designed to furnish the TROs with qualified translational researchers; 3) TROs Lacked sustainable funding sources; 4) TROs lacked robust management structures.

Most TROs were launched by bio-medical research institutions and are characterized as actual clinic research centers. Although there are some significant translational research occurring outside of government support, such as industries (e.g., pharmaceutical companies, CROs) and foundations/NGOs [42], internationally, governments plays great role in planning, organizing and funding translational research. In the US, National Institutes of Health (NIH) launched the Clinical and Translational Science Awards (CTSA) and established the National Centre for Advancing Translational Sciences (NCATS) [43]. Government establishments such as the Medical Research Council (MRC) in the UK [44,45], the French National Research Agency (ANR) [46], the National Health and Medical Research Council (NHMRC) in Australia [47], and the Canadian Institutes for Health Research (CIHR) [48] guided translational medicine in these countries. In China, the government encouraged construction of TROs, and a number of TROs have been established since 2009 [49]. Many hospitals are setting up translational medicine centers without the necessary infrastructures, robust management systems and government support that characterize TROs in high income countries [22]. China lacks a national systematic plan and a national translational research center to build a roadmap for deployment of effective translational medicine. We found that most Chinese TROs were launched by groups of research institutions without specific government participation in organizing, funding, and coordinating although all of TROs were established in public institutions which have significant government support for their activities. TROs, as multidisciplinary entities, offer the potential to bring together different perspectives to address otherwise intractable problems [50]. Clinical and Translational Science Centers (CTSCs) in the U.S. widely involve institutes and disciplines, and the various CTSA programs throughout that country are all closely linked [51]. The CTSCs incubate multidisciplinary, cooperative programs that include translational technologies, resources, and novel methodologies; biostatistics; study design and research ethics; participant and clinical interaction resources; community engagement, education, regulatory knowledge and support; informatics; and pilot and collaborative studies [51]. However, we report that TROs in China are characterized as clinical research centers. In addition, most TROs just translate basic research outcomes to clinical practice However, very few TROs involved disciplines related to public health, education, ethnicity, and sociology; engaged the community; or provided training for translational researchers.

### Current educational programs are not providing qualified translational researchers for TROs

The importance and difficulties of recruiting, mentoring, and retaining an international assembly of qualified clinical and translational scientists has been recognized [52]. Addressing the nurturing of translational investigators, the Association of American Medical Colleges convened its second Clinical Research Task Force (CRTF II) in 2006 and underscored the importance of requiring all future physicians to be educated in the principles and methodologies of translational and clinical research [53]. CRTF II suggested a degree program (Master’s level training at a minimum) [53]. Many CTSC programs provide PhD and Master's degrees [54-56]. In addition, CRTF II suggested start-up support for junior translational investigators, accelerated training, and modifying training (K23 and K24) awards to nurture translational investigators [53].

Translational medicine is likely to have a profound impact on future medical education in China, and the purposeful training of translational research will need to be developed [22]. We report that although most of the TROs had the objective of education, the concept of multidisciplinary researcher cultivation was limited to basic and clinical medicine instead of broader disciplines such as sociology, management, law, public health, and social sciences. What’s more, China has no Master's or PhD degree programs in translational medicine to date.

### TROs Lacked sustainable funding sources

Successful translational research will require substantially more money, an important part of which is government funding. For example, CTSAs provided $500 million annually to 60 translational medical centers throughout the U.S. [6]. The MRC UK committed £250 million to deliver this important part of its mission [44]. In addition, CTSCs attract funds from enterprises and social agencies [57]. In China, government increased and is increasing investment to translational research; the National Natural Science Foundation of China approved a budget of 18.27 billion RMB (approximately 2.9 billion USD) for fiscal year 2011 for translational research, which was an 89% increase from 9.65 billion RMB (approximately 1.52 billion USD) from fiscal year 2010. Recently, translational research has been included in certain government projects, such as the National High Technology Research and Development Program (863) and the National Basic Research Program (973) [22]. Projects with potential translational research are strongly encouraged and have priorities over more speculative applications [28,58]. The government is making investments into organizations that focused on translation such as Beijing Genomics Institute (BGI) [59] and Genzyme of Cambridge, Massachusetts and Tianjin International Joint Academy of Biotechnology and Medicine (TJAB)) [60]. However, this study found that in at least these 8 TROs, funds mainly came from research grants or partners of TROs. The government allocated little special funds to TROs construction or researcher cultivation as CTSAs did. Besides, social entrepreneurship is relatively new in China and its concept was first introduced through various symposiums and conferences in 2004. In 2012, 54% of surveyed social enterprises in China are under 3 years old, of which 21% are less than 1 year old, and 38% were older than 5 years. Compared to 2011 survey, the number of mature social enterprises increased from 15% in 2011 to 38% in 2012 [61]. Thus, TROs in China seldom attract funds from social enterprises or donors. Therefore, a sustainable funding mechanism is one major challenge facing translational research in China.

### Chinese TROs lacked appropriate management

The translational continuum is a complicated process, including many steps and involving multidisciplinary research teams that interact with each other across the continuum [62,63]. Management in TROs has the function of obtaining funding, monitoring, guiding and providing shared sources, promoting multidisciplinary collaboration/cooperation for translational research [64]. To meet the needs of translational medicine, current scientific research management teams should adapt to the tenets of translational research [22]. For example, the centralization of CTSCs in the U.S. is one way to remove organizational barriers, and 58% of CTSCs are moving toward administrative centralization [53]. A flexible framework for performance assessment that tracks progress and incentivizes fruitful activities is very important for cost-effective translational research [64]. However, there is no consensus on the methodologies or frameworks for assessing TROs performance [65]. Although we indicated that most TROs in China had established management teams, specific measures in the following areas were still lacking: developing and nurturing researchers, promoting multidisciplinary cooperation, conducting performance assessments, and offering incentives for partners. TROs also lacked mechanisms for profit- and property-sharing, cost-effectiveness assessment systems across different stages in the translational research continuum, and conceptions of “providing service.”

### Implications for practice and research

Results of this study had the following implications for future practice in China.

### TROs in China need to increase multidisciplinary cooperation

Multidisciplinary research teams in translational research span the life sciences, social sciences, and the physical sciences. The recruitment of sufficient and appropriate participants for translational medicine requires TROs to collaborate with their communities [53]. Furthermore, translational medicine entails not only a “from-bench-to-bedside” approach but also preventive medicine and public policy [17,18]. Community engagement and the broadening of disciplines and research teams in social sciences, physical sciences, health policy, and public health should be emphasized by Chinese TROs.

### Medical education needs innovation

Translational researchers must have multidisciplinary knowledge and capability [66].Translational researchers need to have the collaboration skills to allow ensure clinicians and scientists to function in effective inter-professional, multidisciplinary teams [67]. Therefore, current medical education program must be tailored towards training multidisciplinary translational researchers in order to achieve the full benefits and promising applications of translational medicine [68]. China has begun cultivating interdisciplinary talent recently; this cultivation has developed slowly [69]. Although present medical education programs, including 8- and 5-year programs, have the potential to cultivate medical students with both clinical research and basic research [28], they cannot provide multidisciplinary knowledge wider than medical knowledge because, in the traditional Chinese university model, colleges of different disciplines seldom communicate with one another [22]. Therefore, current medical education needs innovation, such as launching Master's or PhD degrees in translational medicine by the Ministry of Education which emphasize professional incubation to generate future-leading, multidisciplinary translational scientists in TROs.

### Exploring appropriate management of TROs is of crucial importance

Effective management can ensure the overall excellent performance of TROs [53,70]. Translational research is particularly complex and requires a scalable and adaptive management approach [71]. At the early stages of Chinese TROs, it is particularly important to explore management models that include creating a “culture” of multidisciplinary cooperation in TROs, medical schools and their affiliated teaching hospitals; construct a flexible performance assessment system that considers both translational research and academic recognition in researchers' performance assessments; and explore sustainable funding resources.

### Translational research in health policy should be emphasized in China

As in most countries, health research and policy making in China usually operate in separate environments, each with its own professional culture, and most public health research stops at publication [71]. As a result, many public health policies in China are not based on high-quality evidence. A platform using TROs for bilateral communication between researchers and policy makers is needed to improve mutual understanding and to establish an effective and efficient dialogue channel. Translational research in health policy that can promote evidence-based health policies [18,71] should be underscored in China.

### Strengths and limitations

A major strength of the case study approach is the opportunity to use multiple sources of evidence such as interviews and document reviews [32]. The main limitation is that we collected data about some cases indirectly through conferences, reports, and literature, some of which may have been incomplete. A qualitative multiple case study method was used to collect data, which as a form of qualitative descriptive research, looks intensely at an individual or small participant pool, drawing conclusions only about that participant or group and only in that specific context. In this case, we did not focus on the discovery of a universal, generalizable truth, nor did we look for cause-effect relationships; instead, we placed emphasis on exploration and description [72].

## Conclusions

Establishing TROs is an important approach in promoting tranlationational medicine. TROs should be cooperative platforms, centers for human resource cultivation (translational researchers) and agencies of social services (consulting and training) and translational research. Translational medicine in China is developing quickly, and many TROs have been established throughout the country. However, while these TROs maintain key staff (academic, program and administration, technical, and research), headquarters and office accommodation, and suitable experimental research facilities, they still face many challenges. Therefore, it is of paramount importance to strengthen the government's role in widening multidisciplinary cooperation, promoting translational researcher cultivation, and designing management approaches that foster an open culture of cooperation.

## Abbreviations

HIV: Human immunodeficiency virus; ICTM: International Conference on Translational Medicine; U.S.: The United States; SAS-CTM: Sino-American Symposium on Clinical and Translational Medicine; TROs: Translational research organization; PUMCH: Peking Union Medical College Hospital; TMC: Translational medical center; CTMR: College of Translational Medical Research; TRCSR: Translational Research Center of Stem Cell and Regenerative Medicine; NIH: National Institute of Health; CTSA: Clinical and Translational Science Awards; NCATS: National Centre for Advancing Translational Sciences; MRC: Medical Research Council; ANR: National Research Agency; NHMRC: National Health and Medical Research Council; CIHR: Canadian Institutes for Health Research; MDRTs: Multidisciplinary research teams; STSI: Scripps Translational Science Institute; CRTF II: Second Clinical Research Task Force.

## Competing interests

The authors declare that they have no competing interests.

## Authors’ contributions

CL designed the study. LZ and YL designed the data collection instrument and collected. LZ and YL analyzed the data. YL drafted the manuscript. HB commented, edited and revised the final draft. JE provided guidance on design and analysis, provided critical review, edited, and revised the manuscript. All authors read and approved the final manuscript.

## Supplementary Material

Additional file 1 Summary of themes and related categories. Click here for file
