# Supplementary material for: Challenges facing translational research organizations in China: a qualitative multiple case study
Source: J Transl Med. 2013 Oct 13;11:256. doi: 10.1186/1479-5876-11-256 (PMC3853359; doi:10.1186/1479-5876-11-256)
Supplement: Additional file 1 — Summary of themes and related categories. [file 1479-5876-11-256-S1.docx]

**Appendix 1 Summary of themes and related categories**

| Theme | Categories |
| --- | --- |
| Objectives, sponsor and Funding | 1. Objective 2. Organizer 3. Funding source |
| Partners and research teams | 1. Partners 2. Multidiscipline research team |
| Management | 1. Management team 2. Multidiscipline research team management (recruitment, cultivation and performance evaluation) 3. Research project management 4. External and internal cooperation management 5. Fund management |
| Achievement | 1. Infrastructure establishment 2. Translational research 3. Cultivation of translational researchers 4. Service 5. outcomes (publication, awards, intellectual property and products; and international communication) |
